# Clustering of End Stage Renal Disease Patients by Dimensionality Reduction Algorithms According to Lymphocyte Senescence Markers

**DOI:** 10.3389/fimmu.2022.841031

**Published:** 2022-05-09

**Authors:** Georgios Lioulios, Asimina Fylaktou, Aliki Xochelli, Erasmia Sampani, Ioannis Tsouchnikas, Panagiotis Giamalis, Dimitra-Vasilia Daikidou, Vasiliki Nikolaidou, Aikaterini Papagianni, Ioannis Theodorou, Maria Stangou

**Affiliations:** ^1^ Department of Nephrology Aristotle University of Thessaloniki, Hippokration Hospital, Thessaloniki, Greece; ^2^ Department of Immunology, National Peripheral Histocompatibility Center, Hippokration Hospital, Thessaloniki, Greece; ^3^ Laboratoire d’Immunologie, Hôpital Robert Debré, Paris, France

**Keywords:** dialysis, ESRD, hemodiafiltration, machine learning, immune senescence, immune exhaustion

## Abstract

End stage renal disease (ESRD) engenders detrimental effects in the Immune system, manifested as quantitative alterations of lymphocyte subpopulations, akin, albeit not identical to those observed during the ageing process. We performed dimensionality reduction of an extended lymphocyte phenotype panel of senescent and exhaustion related markers in ESRD patients and controls with Principal Component Analysis (PCA) and Uniform Manifold Approximation and Projection (UMAP). The plane defined by the first two principal components of PCA showed two fuzzy clusters, for patients and controls, respectively, with loadings of non-senescent markers pointing towards the controls’ centroid. Naive lymphocytes were reduced in ESRD patients compared to controls (CD4+CD45RA+CCR7+ 200(150-328) *vs*. 426(260-585cells/μl respectively, *P* = 0.001, CD19+IgD+CD27- 54(26-85) *vs*. 130(83-262)cells/μl respectively, *P* < 0.001). PCA projections of the multidimensional ESRD immune phenotype suggested a more senescent phenotype in hemodialysis compared to hemodiafiltration treated patients. Lastly, clustering based on UMAP revealed three distinct patient groups, exhibiting gradual changes for naive, senescent, and exhausted lymphocyte markers. Machine learning algorithms can distinguish ESRD patients from controls, based on their immune-phenotypes and also, unveil distinct immunological groups within patients’ cohort, determined possibly by dialysis prescription.

## 1 Introduction

Phenotypic and functional alterations of the immune system occurring with advancing age are commonly attributed to “immune senescence”. This generic term encompasses a wide spectrum of changes including lymphopenia, inverted CD4/CD8 ratio, reduced naive T cells, along with repertoire restriction, proliferative insufficiency, a shift towards more differentiated subsets, dysregulation of apoptosis and increased secretion of growth factors and proinflammatory cytokines (1). In particular, ageing T lymphocytes are characterized by low CD28, high CD57 expression and re-expression of CD45RA+ receptor on CCR7- T cells. Meanwhile, the onset of progressive reduction of IgD and CD27 molecules in memory populations, may indicate a senescent B lymphocyte phenotype (1). The majority of these immunological changes are plausibly anticipated in older individuals, in context of the ageing process; however, their occurrence may be observed in chronic inflammatory conditions as well, in relation to a resultant ambience of premature immune senescence (2–6). Further, chronic inflammatory state may be additionally associated with a distinct T cell phenotype, namely the “immune-exhaustion phenotype”, characterized by increased expression of PD1 (7).

End-Stage Renal Disease (ESRD) may be regarded as essentially a low-grade chronically sustained inflammation state, sharing similarities, and mimicking the effects of the process of ageing on the immune system. Moreover, uremic toxin accumulation and chronic oxidative stress result in an ESRD specific microenvironment potentially facilitating presentation of unexpected and unique T cell phenotypes (8). Clinical consequences of immune senescence include susceptibility to infections, developments of certain malignancies and substantially increased incidence of cardiovascular disease, collectively underlying the increased morbidity and mortality among ESRD patients of all age groups (8).

Phenotypic alterations of immune cells in ESRD, including the expression of surface markers have been previously studied [reviewed in (9)]. Provisional analysis of T cell phenotype in ESRD has documented a senescence resembling T cell phenotype; nevertheless, most of the studies analyzed a restricted number of T cell surface molecules, thus not strong enough to support a definitive “immune-senescent” or “immune-exhausted” phenotype (10–13).

Availability of novel markers remains limited at present, notwithstanding significant scientific advances in the understanding of alterations of lymphocyte quantities and functions, strongly suggesting that ongoing research and updates of the immune phenotyping is mandatory. We endeavor to set out an exhaustive search, using a large panel of T and B cell markers and perform a comprehensive agnostic analysis by unsupervised machine learning techniques, in an attempt to reveal phenotypic patterns of lymphocytes associated with the presence of ESRD

Principal Component Analysis (PCA) is the oldest and simplest unsupervised machine learning method. Initially invented almost a hundred years ago, it still remains a valuable tool, widely used in data processing. PCA allows the visualization of large data tables, by projection onto lower dimensional spaces, and is very helpful in uncovering trends, clusters, and outliers. The data are usually mean-centered and scaled to unit. Individual observations and variables are thus projected in lower dimensional spaces defined by the most important eigenvectors of the covariance matrixes (14). The non-parametric type and its easy interpretation give PCA advantage over more recent dimensionality reduction algorithms.

UMAP, as very recently introduced by McInnes et al. (15), is a novel unsupervised statistical method for projecting multidimensional data onto lower dimensional spaces, but unlike PCA, it is a nonlinear dimensionality reduction technique. UMAP, is a manifold learning algorithm that projects high-dimensional data by transferring them into a lower dimensional space. This algorithm gives higher importance in preserving the local distance of data in space, than long range distances and hence, it achieves an accurate depiction of local data structure.

Machine learning can be used for comprehension and extraction of hidden patterns of complex data matrices such as those produced through immunophenotypic of peripheral blood mononuclear cells (PBMC) with numerous markers.

The purpose of this study was to explore previous concepts collectively and investigate the characteristics of an “ESRD pattern” of lymphocytes, by performing an exhaustive analysis of lymphocyte surface molecules, known to be implicated in ageing and chronic inflammation. We evaluated data by simple unsupervised machine learning techniques, and accordingly, we described distinct groups of ESRD patients based on their immunological profile.

## 2 Materials and Methods

### 2.1 Study Population

This cross-sectional study was performed in a total of 30 adult Caucasian patients (18 females) with ESRD treated with dialysis in the Department of Nephrology, Aristotle University of Thessaloniki, Hippokration Hospital.

#### 2.1.1 Inclusion Criteria

Patients included in the study should be between 18-80 years old, undergoing dialysis for at least 1 year. Dialysis method performed was either Hemodialysis (HD) or Hemodiafiltration (HDF). Choice of the dialysis method was based on the presence of cardiovascular instability, with HDF preferred for those showing either severe hypotensive episodes or cardiovascular disease. All patients should be dialyzed in a regular basis of three times per week, for at least 4hrs, by biocompatible membranes, and receive adequate dialysis, as this was defined by Kt/V > 1.2.

#### 2.1.2 Exclusion Criteria

Patients with comorbid conditions, with potentially immunological implications, were excluded, as well as patients with chronic inflammatory diseases, diabetes mellitus, active malignancy, recent history of infection or vaccination (less than 3 months), and patients receiving immunosuppressive treatment for the past 3 months. Patients who had required transfer from HD to HDF or vice versa in the past 12 months were also excluded.

Twenty healthy volunteers of similar age, sex (7 females), and ethnicity were used as controls.

All study participants (patients and controls) signed an informed consent prior to enrollment. The study was approved by the Institutional Review Board of the Medical School of the Aristotle University of Thessaloniki (ref No 2273/15-12-2020).

### 2.2 Blood Collection and Preparation

Ten milliliters of total blood were drawn from each patient at the beginning of a mid-week routine dialysis session. Samples were collected in EDTA collecting tubes and processed for flow cytometry to evaluate total white cell count, lymphocytes, and subpopulations.

### 2.3 Lymphocytes’ Subset Analysis

Proportions of different CD4, CD8, and B lymphocytes subsets were determined using a cell counter (Navios Flow Cytometer, Beckman Coulter), according to the manufacturer’s recommendations. For each sample four different set of markers were prepared. The lymphocytes were stained with conjugated antibodies for CD45, CD3, CD4, CD8, CD45RA, CCR7, CD28, CD31, CD57, PD1, CD19, CD27, IgD. The antibodies used for the analysis representing specific lymphocyte surface markers, are described in **Table S1**, and different lymphocyte subsets derived from their companied expression are presented in **Table 1**

**Table 1:** Phenotype of T and B cells subsets.

	T cell markers (CD4+, CD8+)	B cell markers (CD19+)
**Naive**	CD45RA+CCR7+	IgD+CD27-
**RTEs**	CD45RA+CD31+	
**Central memory**	CD45RA-CCR7+	
**Effector memory**	CD45RA-CCR7-	
**TEMRA**	CD45RA+CCR7-	
**IgM memory**		IgD+CD27+
**Switched memory**		IgD-CD27+
**Late differentiated**	CD28-CD57+	IgD-CD27-
**Exhausted**	PD1+	

The gating strategy used to define the lymphocytes subsets is shown in **Figure S1**. Based on existing literature, we segregated and demarked CD4 and CD8 T lymphocyte subsets as below: naïve (CD45RA+CCR7+), central memory (CD45RA-CCR7+), effector memory (CD45RA-CCR7-) and effector memory re-expressing CD45RA – TEMRA (CD45RA+CCR7-). We also considered as recent thymic emigrants (RTEs) T cells bearing the phenotype CD45RA+CD31+. Terminally differentiated cells were determined as CD28-CD57+ both in CD4 and CD8 T cells subsets. Finally, PD1+ T cells were considered as exhausted.

Before B cells detection, the cells were washed twice with phosphate-buffered saline (PBS), to remove soluble IgD. In the B cells subsets, we studied four subpopulations: naïve B cells (IgD+CD27-), IgM memory B cells (IgD+CD27+), switched memory B cells (IgD-CD27+) and late memory B cells (IgD-CD27-). Absolute numbers of lymphocyte subpopulations were calculated by using the percentages obtained by flow cytometry and the lymphocyte number from the same sample.

### 2.4 Statistics

Statistical analysis was performed in two steps. First, we used dimensionality reduction algorithms in order to capture meaningful properties of immune senescence related markers in the cohort. PCAs were performed in R with the Factominer package (16). The variables in PCAs were mean centered and scaled to unit. The number of PCs analyzed was based on the corresponding scree plot. We selected the PCs included in the steep part of the eigenvalues plot. UMAP was performed in R with the UMAP package (15). In order to increase the validity of the method and ensure reproducible results we run the UMAP algorithm twice, slightly altering settings. The second step is a classic hypothesis testing to evaluate differences on markers between groups suggested by PCA biplots and UMAP projected ellipsoids. Inferential statistical analysis was performed with SPSS.25 IBM Corp, Armonk NY for Windows. Continues variables were checked for normality distribution by Shapiro-Wilk and/or Kolmogorov-Smirnov tests and expressed as Mean ± Standard Deviation or Median (interquartile range). As all variables, except for age, were non-parametric, differences between two variables were estimated by Mann-Whitney-U test. Kruskal-Wallis test followed by Bonferroni correction, was applied to compare data among more than two variables. Finally, chi-square test was used for categorical variables. *P* values < 0.05 were considered statistically significant.

## 3 Results

### 3.1 Patients’ Characteristics

Patients’ characteristics are shown in **Table 2**. Mean age of the patients was 58 ± 15 years, similar to healthy controls 53 ± 13 years, *P* = 0.28, and mean dialysis vintage was 80 ± 50 months.

**Table 2:** Patients’ characteristics.

	All patients	HD	HDF	p (HD *vs*. HDF)
n	30	19	11	
Age (yrs)	58 (47.5-71.5)	65 (55-77)	53 (39-60)	0.028
Female (n)	13	10	3	0.17
Dialysis vintage (months)	78.8 (41-101)	62 (21-85)	96 (77-169)	0.005
History of transplantation (%)	5 (16.7)	2 (10.5)	3 (27.2)	0.23
**Primary disease**				
Primary Glomerulonephitis (%)	7 (23.3)	4 (21.1)	3 (27.2)	0.69
Polycystic disease (%)	5 (16.6)	2 (10.5)	3 (27.2)	0.23
Unknown (%)	11 (36.6)	9 (47.3)	2 (18.2)	0.10
Reflux Nephropathy (%)	4 (13.3)	2 (10.5)	2 (18.2)	0.55
Other (%)	3 (10)	2 (10.5)	1 (9)	0.89
**Comorbidities**				
Hypertension	15 (50)	9 (47.4)	6 (54.5)	0.705
Cardiovascular	6 (20)	6 (31.6)	0 (0)	0.037
Hyperparathyroidism	19 (63.3)	11 (57.9)	8 (72.7)	0.417
**Medication**				
Epoetin (iu/wk)	3500 (0-6000)	6000 (2500-9000)	0 (0-4500)	0.03
Iron (mg/month)	150 (75-150)	200 (100-300)	100 (0-200)	0.041
Paricalcitol (mg/wk)	0 (0-7.5)	0 (0-6.25)	5 (0-10)	0.10
**Dialysis Membranes**				
PEPA		0 (0)	7 (63.6)	
PES		9 (47.4)	0 (0)	
PES/PVP		2 (10.5)	0 (0)	
PES/PVP/PA		4 (21.1)	0 (0)	
PS		0 (0)	4 (36.1)	
PS (Helixone)		4 (21.1)	0 (0)	
Volume of HF (lt)		N/A	19.9	
				
**Laboratory parameters**				
WCC (cells/μL)	7050 (5475-7525)	6800 (5200-7500)	7100 (5500-8300)	0.42
Neutrophils (cells/μL)	4400 (3575-5400)	4400 (3600-5300)	4600 (3400-5800)	0.672
Lymphocytes (cells/μL)	1500 (1300-1800)	1500 (1100-1800)	1400 (1400-1800)	0.699
Monocytes (cells/μL)	587 (533-681)	592 (547-684)	540 (504-656)	0.328
Ht (%)	35.1 (33.5-38.1)	35 (33.2-36.4)	35.6 (33.9-39.7)	0.287
Hb (g/dL)	11.4 (11-12.3)	11.4 (11-11.8)	11.8 (11.2-13.2)	0.250
Platelets (10^3^/μL)	219 (181-268)	228 (174-350)	211 (200-262)	0.672
Serum Urea (mg/dl)	123 (109-148)	121 (107-137)	147 (119-163)	0.735
Serum Creat (mg/dl)	9 (6.8-11)	8.3 (5.8-9.5)	12.1 (10.2-12.6)	<0.001
CRP (mg/L)	2.1 (1.3-7.5)	2.9 (1.6-8.2)	1.7 (1.1-7.3)	0.42
Serum Albumin (g/dL)	4 (3.9-4.2)	4.1 (3.9-4.3)	4 (3.8-4.2)	0.395
Ca (mg/dl)	9.2 (8.8-9.8)	9.2 (8.9-9.8)	9.2 (8.8-9.9)	0.8
P (mg/dl)	4.3 (4.3-4.9)	4 (3.3-4.4)	4.7 (4.3-5.4)	0.023
iPTH	232 (104-385)	223 (105-374)	361 (102-412)	0.525
Ferritin	243 (184-439)	291 (200-450)	301 (108-463)	0.641
C3	77 (69.5-86.8)	83.8 (68.3-89.7)	76 (72-84)	0.42
C4	25 (21.9-29.1)	27 (23.3-34.5)	22.5 (17.9-26.5)	0.07

HD, Hemodialysis; HDF, Hemodiafiltration; PEPA, Polyester/Polymer; PES, Polyethersulfone; PVP, Polyvinilpyrrolidone; PA, Polyamide; PS, Polysulfone; HF, Hemofiltration.

Neutrophil count and their percentage on white cell count were significantly higher in ESRD patients compared to healthy controls [4400(3575-5400)cells/μl *vs* 3450(2925-4175)cells/μl, *P* = 0.008, 62.5(57.8-70.9)% *vs* 56.2(55.5-61.2)%, *P* < 0.001, respectively], while lymphocytes count and percentage were significantly reduced [1500(1300-1800)cells/μl *vs* 2250(1600-2575)cells/μl, *P* < 0.001, 19.6(15.4-23.9)% *vs* 26.1(21.6-33.7)%, *P* = 0.001, respectively]. Monocytes’ count was increased in ESRD patients [587(533-682)cells/μl *vs* 489(451-595)cells/μl, *P* = 0.029]. Total count of white cells, as well as, eosinophils, basophils, platelets and monocytes percentage did not differ between the two groups.

### 3.2 PCA Projections of Lymphocyte Subsets in ESRD Patients and Controls

**Figure 1:**
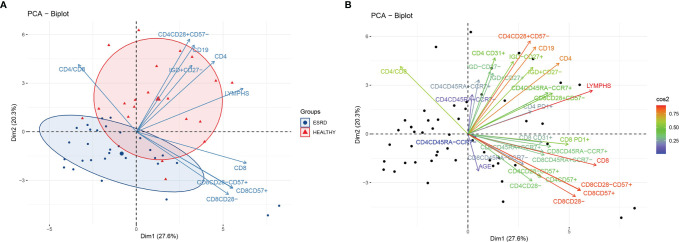
**(A)** PCA biplot of immune senescence markers of ESRD patients and healthy controls, on a plane defined by the first two eigenvectors of the covariance matrix, separation of the two populations. Triangles represent healthy individuals and circles ESRD patients. Large symbols represent the centroid of each population and ellipses represent gaussian kernel density estimates for each class. Only the ten variables with the largest loadings are depicted. **(B)** PCA biplot of immune senescence markers of ESRD patients and healthy controls on a plane defined by the first two eigenvectors of the covariance matrix. Points represent individuals and arrows the corresponding variables. The color of each arrow is proportional to the cos2 of the explained variance according to the color vector on the right side of the figure.

We initially performed a PCA, applied to both patients and controls, and included all the variables from the dataset of flow cytometric analysis. The PCA plot is depicted in **Figure 1**. PC1 and PC2 explain 46.5% of total variance. The centroids and the corresponding ellipses of patients and control groups projected on this first two eigenvector delimited plane are far apart from each other suggesting that these two populations form two distinct clusters with limited admixture between the patients and controls in this space (**Figure 1A**). Moreover, we observe a clustering of variables in two main beams, one of which, with direction to the upper right quadrant, points to the centroid of the control group, while the second one appears to be orthogonal to the first (**Figure 1B**). Projections on the second and third eigenvector delimited plane did not show a clear distinction between patients and controls (data not shown). Notably, the loading representing age of patients was rather short, indicating the low contribution of this variable to the total variance in this plane (**Figure 1B**)

**Figure 1A** also shows the first two eigenvector delimited plot by representing only the ten variables with the highest explained variance according to the loading plot (**Figure S2**). CD4+, CD4+CD28+CD57-, CD19+ and CD19+IgD+CD27- are the main variables directing towards the controls’ centroid, and opposite to the patients’ centroid. Inferential statistics performed for these PCA-indicated variables, revealed several significant differences between ESRD patients and controls, and are described in **Tables 2**, **3**. ESRD patients were characterized by severe lymphopenia [1500(1300-1800) *vs* 2250(1625-2250)cells/μl, P < 0.001] as well as decrease of all ‘immature’ and less-differentiated T cell subsets, predominantly affecting the CD4 compartment. RTEs, Naïve and central memory CD4 cells, were also severely reduced in ESRD compared to controls, while CD4+CD28- cells were significantly increased, despite the general CD4 lymphopenia. Such differences were not encountered for CD8 positive cells, where the most important alteration affected PD1 expression, resulting to a significant elimination of CD8+PD1+ cells (**Table 3**).

**Table 3:** Differences in the phenotypic pattern of T cell population between ESRD patients and healthy controls.

	ESRD	Controls	*P*		ESRD	Controls	*P*
n	30	20			30	20	
*CD4 subsets* (cells/μl)				*CD8 subsets* (cells/μl)			
CD4+	693 (483-815)	1002 (916-1306)	**<0.001**	CD8+	356 (230-608)	470 (355-826)	0.08
CD45RA+CCR7+	200 (150-328)	426 (260-585)	**0.001**	CD45RA+CCR7+	147 (59-249)	158 (94-332)	0.41
CD45RA-CCR7+	351 (271-498)	591 (476-722)	**0.001**	CD45RA-CCR7+	58 (15-102)	137 (18-218)	0.12
CD45RA-CCR7-	10 (5-18)	9 (1-24)	0.54	CD45RA-CCR7-	19 (1-64)	17 (1-73)	0.93
CD45RA+CCR7-	16 (7-29)	16 (5-31)	0.86	CD45RA+CCR7-	76 (41-140)	53 (21-145)	0.44
CD45RA+CD31+	127 (87-209)	251 (138-354)	**0.001**	CD45RA+CD31+	159 (91-236)	203 (160-300)	0.17
CD28+CD57-	605 (416-703)	988 (777-1185)	**<0.001**	CD28+CD57-	144 (106-185)	291 (191-279)	**<0.001**
CD28-CD57+	20 (12-47)	25 (4-46)	0.94	CD28-CD57+	105 (33-274)	120 (39-301)	0.6
CD28-	46 (26-104)	31 (11-58)	**0.05**	CD28-	168 (64-406)	155 (67-352)	0.75
CD57+	29 (15-60)	27 (10-52)	0.66	CD57+	113 (39-289)	126 (40-318)	0.69
PD1+	76 (46-109)	91 (56-150)	0.18	PD1+	76 (50-142)	138 (98-260)	**0.004**

Bold values signify statistical significance.

Changes of B cells were even more impressive, with total population and individual subsets being significantly lower in the patients’ cohort compared to controls. This downwards tendency of B cells affected mostly the IgM memory B cell population (IgD+CD27+), as seen by the Bpatients/Bcontrols ratios (**Table 4**).

**Table 4:** Phenotypic differences of B cell compartment, between ESRD patients and healthy controls.

	ESRD	Controls	*P*	B_patients_/B_controls._
n	30	20		
Bcells (cells/μl)				
CD19	85 (68-132)	230 (167-408)	**<0.001**	0.37
CD19+IgD+CD27- (cells/μl)	54 (26-85)	130 (83-262)	**<0.001**	0.41
CD19+IgD+CD27+ (cells/μl)	5 (3-11)	28 (17-44)	**<0.001**	**0.18**
CD19+IgD-CD27+ (cells/μl)	13 (9-19)	45 (25-86)	**<0.001**	0.29
CD19+IgD-CD27- (cells/μl)	6 (4-11)	22 (11-43)	**<0.001**	0.27

Bold values signify statistical significance.

### 3.3 PCA Projections According to Dialysis Modality

**Figure 2:**
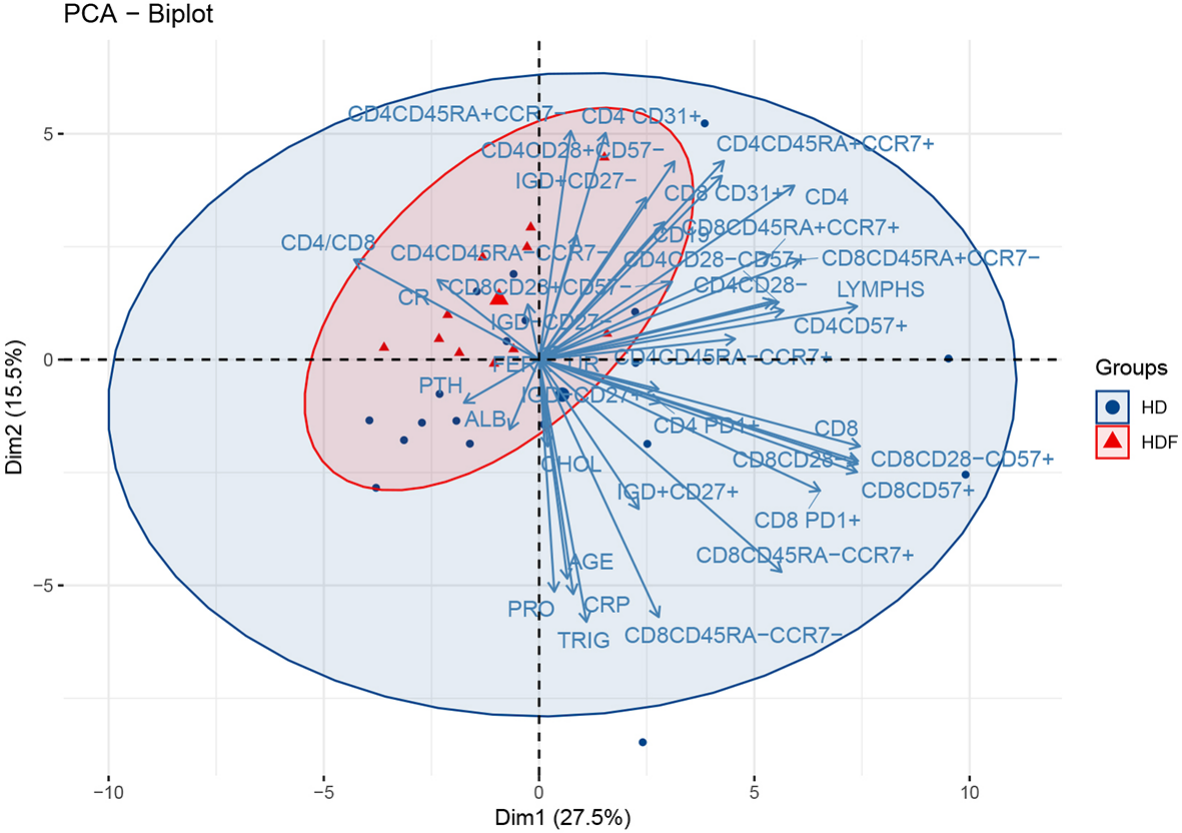
PCA biplot of immune senescence markers and biochemical parameters of ESRD patients, on a plane defined by the first two eigenvectors of the covariance matrix. The loadings represent the corresponding variables. Triangles represent HDF and circles HD patients. Large symbols depict the centroid of each population and ellipses represent gaussian kernel density estimates for each class.

We subsequently attempted to search for further classification of patients, in groups carrying a discrete immune phenotype. We therefore performed PCA restricted to patients’ cohort only, including lymphocyte markers together with certain biochemical indices. The PC1 – PC2 plot which explains 43% of the total variance is seen in **Figure 2**. The present PCA could discriminate between patients on different dialysis methods, namely HD and HDF. However, we could not detect any other clustering of the patients’ cohort, for example based on duration of therapy or primary cause of ESRD (data not shown). HDF patients gather in the upper left quadrant, while the centroid of HD patients lies in the low right quadrant. There seems to be an extensive admixture between the two populations, as HDF patients appear as a subset within the whole patients’ population. The variables cluster in two main beams, one of which points towards the HD patients’ centroid and consists largely of CD8 senescent subsets. The ten variables that mostly contribute to the variance of this PCA are seen in the eigenvalues plot (**Figure S3**). Out of those variables, CD8+CD45RA-CCR7+, CD8+PD1+ and CD8+CD45RA-CCR7- seemed to be most important in discriminating between HD and HDF patients, as their direction almost coincided to the centroids connecting line. Indeed, inferential statistics showed higher levels of these variables in HD compared to HDF patients [60(43-268) *vs* 29(1-87)cells/μl, P = 0.055, 100(52-165) *vs* 69(36-102)cells/μl P = 0.08, 26(5-73) *vs* 8(0-20)cells/μl, P = 0.04, respectively], although not always reaching statistical significance. Of note, PCA also revealed an upwards tendency of CD8 senescent subsets, including CD8+CD28-, CD8+CD28-CD57+ and CD8+CD57+, within HD patients, although not statistically significant, in inferential statistics.

### 3.4 UMAP Projections of ESRD Patients and Controls

**Figure 3:**
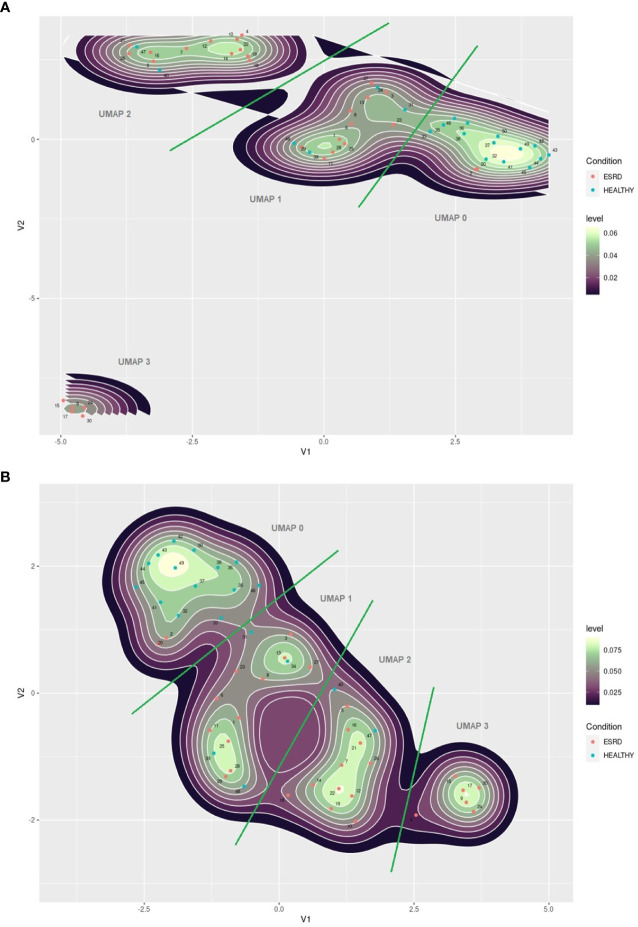
**(A)** UMAP plot of immune senescence markers of ESRD patients and healthy controls, 2 components, 5 neighbors. **(B)** UMAP plot of immune senescence markers of ESRD patients and healthy controls, 2 components, 10 neighbors. The red points indicate ESRD patients and the blue points healthy individuals. Numbers indicate patients ID.

We performed a UMAP based dimensionality reduction on the flow cytometric data of both groups, patients and healthy controls, in an attempt to cluster our observations on the basis of age and immune phenotype only. We ran the algorithm twice; initially with the number of neighbors set to 5, and secondly set to 10. As shown in **Figure 3**, both settings revealed four clusters according to the spatial density estimation of the projected data. The group defined as UMAP 0 mainly included healthy controls, while ESRD patients were not a single group, but rather separated in three clusters, generating three distinct groups (UMAP 1, 2 and 3). We then applied a formal hypothesis testing analysis for all markers between the different groups suggested by UMAP. The results are described on **Table 5**. The main significant differences among UMAP suggested groups were for lymphocyte sub populations but no significant difference for age, sex, dialysis method, or biochemical profiles was found. There is a gradual reduction of total lymphocytes, from UMAP 0 group towards UMAP 3 group, affecting predominantly the CD4+ cells and their subsets. Most interestingly, this reduction was more prominent for naïve CD4+ subsets, CD4+CD45RA+CCR7+, CD4+CD45RA-CCR7+, CD4+CD45RA+CD31+ and CD4CD28+CD57- cells, while senescent CD4 subsets seemed to have no remarkable alterations among UMAP groups.

**Table 5:** CD4, CD8 and B cells subsets count based on clustering of ESRD groups according to UMAP.

	UMAP 0	UMAP 1	UMAP 2	UMAP 3	*P*
n	16	15	14	5	
Age (yrs)	57 (50-64)	51 (33-71)	56 (44-66)	60 (57-80)	0.285
Lymphocytes (cells/μl)	2500 (2175-2675)	1800 (1700-1900)**	1400 (1300-1500)**^††^	900 (750-950)** ^††‡‡^	**<0.001**
***CD4 T subsets* (cells/μl)**					
Total CD4	1217 (677-1398)	797 (724-899)**	658 (575-734)** ^†^	450 (306-471)** ^††‡‡^	**<0.001**
CD45RA+CCR7+	461 (312-603)	317 (197-406)*	209 (176-273)** ^††^	137 (79-179)** ^†‡^	**<0.001**
CD45RA-CCR7+	615 (572-850)	359 (284-528)**	390 (301-473)**	265 (203-293)** ^†‡^	**<0.001**
CD45RA-CCR7-	9 (2-24)	10 (8-28)	6 (2-15)	6 (5-10)	0.215
CD45RA+CCR7-	20 (6-30)	23 (11-69)	9 (5-24) ^†^	14 (6-17)	0.135
CD45RA+CD31+	266 (149-367)	176 (95-263)*	127 (86-212)**	52 (39-111)** ^†‡^	**<0.001**
CD28+CD57-	1107 (897-1219)	698 (650-756)**	578 (500-677)**	310 (263-434)** ^††‡‡^	**<0.001**
CD28-CD57+	27 (5-60)	32 (15-60)	18 (4-35) ^†^	14 (3-19)	0.096
CD28-	36 (15-67)	61 (26-113)	41 (13-69)	35 (14-110)	0.493
CD57+	32 (14-66)	42 (18-66)	23 (8-46) ^†^	21 (4-22)	0.069
PD1+	97 (57-165)	80 (51-118)	82 (64-100)	47 (24-90)	0.240
***CD8 T subsets* (cells/μl)**					
Total CD8	543 (422-933)	581 (386-649)*	260 (203-378)**	148 (124-232)** ^††‡^	**<0.001**
CD45RA+CCR7+	182 (128-391)	243 (84-365)	109 (58-179)* ^†^	73 (28-117)* ^†^	**0.012**
CD45RA-CCR7+	171 (18-361)	41 (10-145)	84 (58-108)	43 (9-50) ^‡^	0.099
CD45RA-CCR7-	35 (1-92)	20 (0-73)	15 (2-35)	23 (1-36)	0.769
CD45RA+CCR7-	99 (30-150)	112 (67-201)	49 (27-81) ^†^	32 (24-75) ^†^	**0.045**
CD45RA+CD31+	203 (163-300)	234 (153-344)	141 (62-184)* ^†^	91 (44-108)** ^††^	**0.002**
CD28+CD57-	295 (208-383)	188 (153-251)*	138 (110-165)** ^††^	87 (68-99)** ^††‡‡^	**<0.001**
CD28-CD57+	191 (52-321)	269 (83-432)	32 (22-130)* ^††^	33 (31-53)* ^††^	**0.001**
CD28-	191 (88-368)	401 (135-493)	119 (49-233) ^††^	60 (51-127)* ^††^	**0.006**
CD57+	191 (58-323)	285 (88-444)	43 (26-136)* ^††^	39 (35-56)* ^††^	**0.001**
PD1+	186 (120-381)	102 (69-165)*	97 (67-114)**	52 (39-65)**	**0.002**
***B cells* (cells/μl)**					
CD19	358 (144-461)	133 (94-186)**	82 (69-113)** ^††^	50 (17-87)** ^††^	**<0.001**
CD19+IgD+CD27-	156 (76-280)	86 (51-115)*	54 (31-73)**	24 (10-64)** ^†^	**0.001**
CD19+IgD+CD27+	28 (17-49)	7 (4-23)**	6 (3-11)**	4 (1-8)**	**<0.001**
CD19+IgD-CD27+	45 (25-102)	16 (13-24)**	13 (10-208)**	7 (4-10)** ^††‡^	**<0.001**
CD19+IgD-CD27-	29 (11-44)	11 (7-14)*	6 (4-10)**	5 (3-10)**	**0.002**

*0.008 < p < 0.05 compared to UMAP 0, **p < 0.008 compared to UMAP 0, † 0.008 < p < 0.05 compared to UMAP 1, †† p < 0.008 compared to UMAP 1, ‡ 0.008 < p < 0.05 compared to UMAP 2, ‡‡ p < 0.008 compared to UMAP 2. Bold values signify statistical significance.

Changes in the CD8 compartment are also interesting. There was a gradual decrease of CD8+ cells from UMAP 0 towards UMAP 3. However, in UMAP 1 group, the predominant CD8+ subsets bared a senescent phenotype. Namely, there are significantly increased counts of CD8+CD28-, CD8+CD57+, CD8+CD28-CD57+, CD8+CD45RA+CCR7- cells, despite the observed lymphopenia. Yet, no significant differences were noticed regarding the naïve CD8 subpopulations. In addition, exhausted CD8 cells also showed substantial deviations among UMAP groups, with increased expression in UMAP 0, eliminating significantly in UMAPs 1-3.

**Figure 4:**
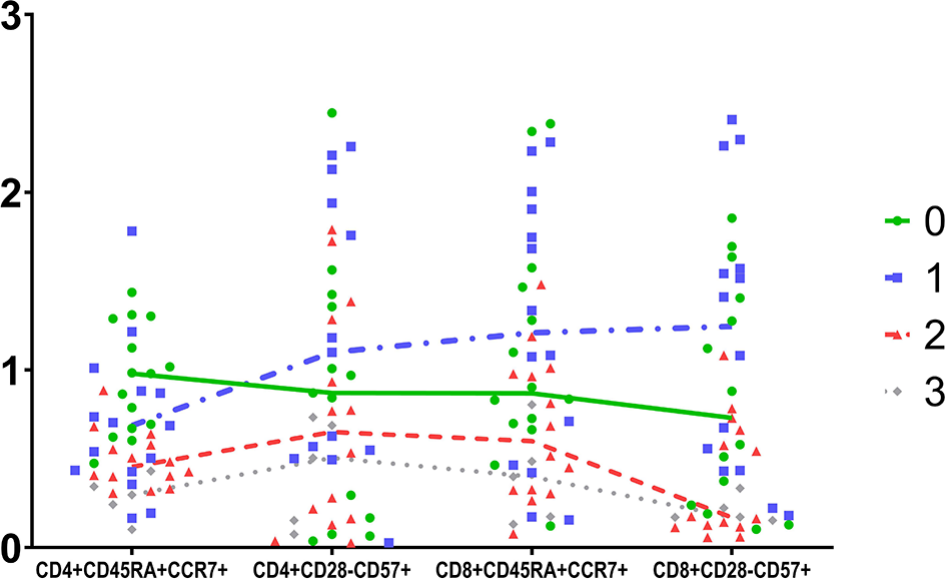
Schematic comparison of four T cells subpopulations in the four groups determined by UMAP. Data were scaled to unit.

UMAP 1 comprised an interesting group of patients characterized by the combination of reduced CD28 and PD1 expression on CD8+ cells, delineating immunologically anergic cells, with reduced capability in the immune synapse. Differences among UMAP groups of patients regarding the senescent phenotypes of CD4 and CD8 cells are schematically depicted in **Figure 4**.

Finally, a significant and gradually deteriorating B lymphopenia was observed in all UMAP groups, compared to controls, mostly affecting the IgM memory subsets of B cells.

Of note, we also ran t-SNE, the findings of which came to agreement with the present UMAP analysis (data not shown).

These disparities lead us to investigate whether those patients exhibit any differences in the dialysis regimen, including time and adequacy of dialysis and type of the dialyzer membrane. For practical reasons, we merged groups UMAP 0 and UMAP 1 to UMAP 0-1, and groups UMAP 2 and UMAP 3 to UMAP 2-3. Comparing dialysis conditions in UMAP 0-1 to UMAP 2-3 patients we found that polysulfone or derivatives used in dialyzer membrane are more frequent in the UMAP 2-3 group compared with the in UMAP 0-1 group (13/17 of UMAP 2-3 patients to 4/13 in UMAP 0-1, *P* = 0.012). No other discrepancies were found in terms of primary disease, immunosuppression history or dialysis modality.

## 4 Discussion

Principal Component Analysis is an unsupervised machine learning technique, which allows dimensionality reduction and visualization of multidimensional datasets. The initial information of the original variables is transformed into a new set of variables called Principal Components (PCs). Each PC explains a certain percentage of variance of the original data. The first PC accounts for the largest variation in the data, followed by gradual decrease of variance explained in each subsequent PC (17).

In this study, we performed a Principal Component Analysis in order to examine whether this simple unsupervised method can unveil trends of the immune phenotype in ESRD patients. Our PCA was indeed able to identify the main lymphocyte phenotypes predominated in ESRD patients, and subsequently differentiate those patients from healthy individuals. In particular, variables with the highest variance explained with the first PCA were clearly clustered in two beams of arrows. Main variables included in the first beam were CD4+, CD4+CD28+CD57-, CD19+, CD19+IgD+CD27-, with their corresponding loadings pointing towards the healthy population, indicative of their preponderance within healthy controls compared to ESRD patients. On the other hand, CD8+, CD8+CD28-, CD8+CD57+ and CD8+CD28-CD57+, consisted the second beam, which appeared parallel to PC1 with a direction in between the ESRD patients and healthy individuals’ group. These latter variables represent the CD8 senescent subsets, and based on their orientation in the PCA, we would not anticipate significant differences between patients and controls, regarding the CD8 compartment. It is of a great interest, that despite that the examined markers are closely related to aging, the loading representing age was rather short in the first two PCs delimited plane. Moreover, the direction of the corresponding arrow does not indicate a close relationship of age to other lymphocyte subsets. This finding might suggest that age may play a minor role to immune alterations in ESRD, in comparison to other factors discussed below.

The novelty of the present study consists on the extended evaluation of lymphocyte markers on ESRD patients, and the agnostic analysis of many lymphocyte subpopulations defined by a large number of markers. Previous studies have described naïve T cell lymphopenia in ESRD, which could be possibly attributed to impaired thymus function and reduced production of immature T cells (18). All these studies however, including our own previous findings, focused on a specific lymphocyte subset, or a restricted number of well-defined subsets. To our knowledge, this is the first time a certain combination of distinct events ensuing ESRD is described; that is severe reduction in naïve CD4+ and B lymphocytes, resulting in substantial lymphopenia. Overall, this first PCA showed a clear difference between ESRD patients and controls, based on naïve CD4 and naïve B cell phenotypes, particularly CD4+CD28+CD57- and CD19+IgD+CD27-, while the more senescent T cells did not differ significantly.

A further aspect uncovered by this PCA is the redundancy of information received when phenotyping such populations. **Figure 1** illustrates some cell populations, represented by loadings that substantially coincide. For instance, CD8+CD28-, CD8+CD28-CD57+, and CD8+CD57+ loadings have approximately the same length and direction, clearly indicating that they represent a single, approximately identical cell subset. Advanced T cell differentiation is characterized by loss of CD28 and express of CD57 molecule (19). According to our results, the two procedures seem to happen simultaneously, as CD8+CD28- cells also expressed CD57, and vice versa, CD8+CD57+ cells lack the CD28 molecule. Therefore, the use of one of these markers, CD28 or CD57, instead of both, on CD8+ cells, might not result in loss of a great amount of information.

Subsequently, PCA was performed in the patients’ group only, in order to scrutinize differences based on their immune phenotype. This PCA shows that the centroids corresponding to HD and HDF modalities lay far apart from each other on the plane although the corresponding ellipses overlap. Particularly, there was a clear preponderance of senescent CD8 subtypes in HD patients compared to those on HDF.

HDF is the dialysis method combining diffusion and convection and achieving a higher removal rate of uremic accumulations, including β2-microglobulin, leptin, Fibroblast Growth Factor (FGF)23, κ and λ light chains and advanced glycosylation end products (20–23). HDF may be related to additional clinical benefits, namely better hemodynamic stability (24), amelioration of systemic inflammation (25), dialysis related amyloidosis (26) and, foremost, an improvement of all-cause mortality, especially in those patients receiving higher volumes of hemofiltration (27, 28). A favorable effect of HDF in immune function has been demonstrated in the clinical setting in a study showing a better response of patients on HDF after influenza vaccination (29). Despite the widespread use and the remarkable clinical benefits, no data are currently available regarding its long-term immunomodulatory effects. Based on the well-established clinical benefits and the differences in uremic toxin removal capacity between HD and HDF, we decided to analyze the immune cell phenotypes in both methods. Our results showed that HDF patients comprise an immunologically distinct population among dialysis patients, featuring lymphocyte phenotypes certainly different from those on HD. The most important deviations were observed in the CD8 compartment, in particular a shift of CD8 senescent subtypes, CD8+CD57+, CD8+CD28-, CD8+CD28-CD57+ and CD8+CD45RA-CCR7-, and CD8 exhausted cells, CD8+PD1+, towards HD patients. It is worth noticing, however, that the two patients’ cohort were not completely identical compared to each other, as shown in **Table 2**. Patients on HDF appeared to be statistically significantly younger than patients on HD. Nonetheless, this difference is not that important to affect immunological parameters, as both cohorts’ median age lies in between sixth to seventh decade of life. Moreover, HDF patients are on dialysis for longer. However, the expected longevity of dialysis therapy is per se an indication for HDF choice.

To our knowledge, there are no studies in the literature which indicate impact of the dialysis techniques in the immunological profile of patients. The clinical consequences of these completely novel findings, though not studied yet, are expected to be extremely important, as increased morbidity and mortality of ESRD patients are integrally connected to alterations of the immune function.

These results, as they raised from PCA on ESRD patients, are indicative of the presence of different immunological groups within the patients’ cohort. Certain discrepancies may follow dialysis prescription, as described above, however, the multiple contribution of several environmental factors, may suggest the presence of further immune deviations. Thus, we performed a further analysis, using UMAP, a non-linear dimension reduction algorithm, to search whether the patients’ group expressed a single immune phenotype or was divided in distinct immune phenotype patterns. We ran the algorithm twice, with different settings to increase the validity of the method. Both analyses resulted in almost identical results. We revealed four clusters, one of which included mostly healthy individuals, while patients were distributed in the rest three clusters. The classification supports the clear distinction of the healthy control group, but also divided ESRD patients into three immunologically different groups. Inferential statistics was performed to estimate differences between groups. The emerging clusters had clear discrepancies in the expression of senescent associated surface markers. UMAP 1 group which showed the most important changes, was characterized by rise of CD8 senescent and anergic subsets, despite the significant lymphopenia, which could be exclusively attributed to the significant reduction of naïve CD4 cells. The UMAP 2 and 3 groups showed further reduction of naïve CD4 subsets, accompanied by fall of the CD8 naïve and also less senescent CD8 subsets. Exhausted CD4 T cells did not differ among groups, while exhausted CD8 T cells were reduced in UMAP groups 2 and 3. This analysis by applying UMAP method revealed some new “hidden” groups of ESRD patients, based on their naïve, senescent, and exhausted subsets of lymphocytes.

Evaluation of parameters that could probably be implicated in this differentiation, revealed that the type of dialyzer membranes may affect the patients’ immune profile. Contact of blood with dialysis materials, during the dialysis session, have been suspected to cause immune phenotype alterations (30), although, no such a relationship has been proved, so far. Our study is the first one to indicate that there might be, indeed, a causative connection between dialysis membranes and immune alterations, however these findings need further evaluation. Alterations of the immune system in end stage kidney disease is a challenging phenomenon with multiple facets and unpredictable implications. Simple unsupervised machine learning methods can facilitate the understanding and visualization of these disturbances. Our study achieved to determine well-defined immune patterns in these patients and delineate the combination of markers, useful in their description. Despite the low number of participants, we could clearly describe a reduction of CD4 naïve subsets, CD19+IgD+CD27+ cells, and also reduction in CD8+PD1+ cells, together with a tendency towards more senescent subsets in ESRD patients. Even more intriguingly, significant connections of immune changes with dialysis methods and dialyzers were revealed and described. Further studies are required to consolidate our findings and clarify yet unknown causes of these alterations. Training of algorithms based on the emerging results, could enable the classification of patients with supervised machine learning techniques, and lead to useful conclusions in the clinical setting.

## Data Availability Statement

The raw data supporting the conclusions of this article will be made available by the authors, without undue reservation.

## Ethics Statement

The studies involving human participants were reviewed and approved by Institutional Review Board of the Medical School of the Aristotle University of Thessaloniki. The patients/participants provided their written informed consent to participate in this study.

## Author Contributions

MS, AF, and GL: conception and study design; ITh and AP: manuscript revision and approval; AF, AX, and VN: laboratory processing; ITs, PG, DD, ES, AF, and GL: data collection and interpretation; GL and MS: manuscript drafting. All authors contributed to the article and approved the submitted version.

## Funding

GL received a grand from the Hellenic Society of Nephrology in order to conduct the present study (Grant No: 48-26/5/2021).

## Conflict of Interest

The authors declare that the research was conducted in the absence of any commercial or financial relationships that could be construed as a potential conflict of interest.

## Publisher’s Note

All claims expressed in this article are solely those of the authors and do not necessarily represent those of their affiliated organizations, or those of the publisher, the editors and the reviewers. Any product that may be evaluated in this article, or claim that may be made by its manufacturer, is not guaranteed or endorsed by the publisher.
