# Supplementary figures and images for: Clustering of End Stage Renal Disease Patients by Dimensionality Reduction Algorithms According to Lymphocyte Senescence Markers
Source: Front Immunol. 2022 May 9;13:841031. doi: 10.3389/fimmu.2022.841031 (PMC9126282; doi:10.3389/fimmu.2022.841031)

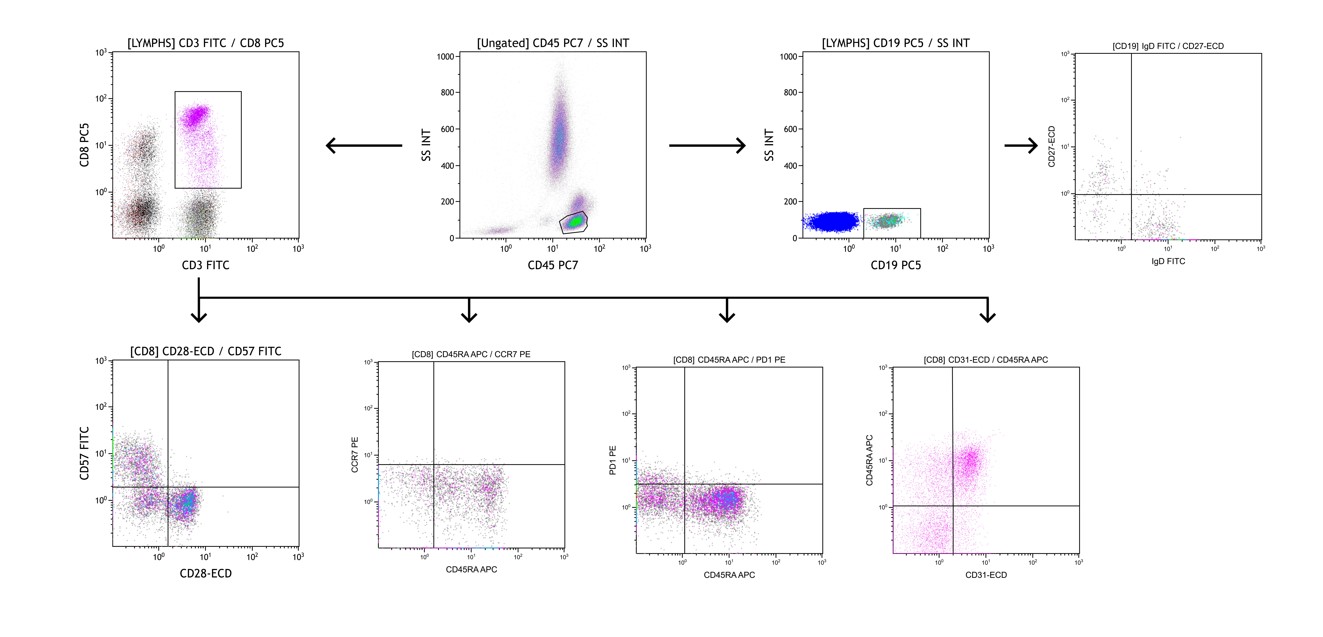

Supplement: Supplementary file 1 [file Image_1.jpeg]

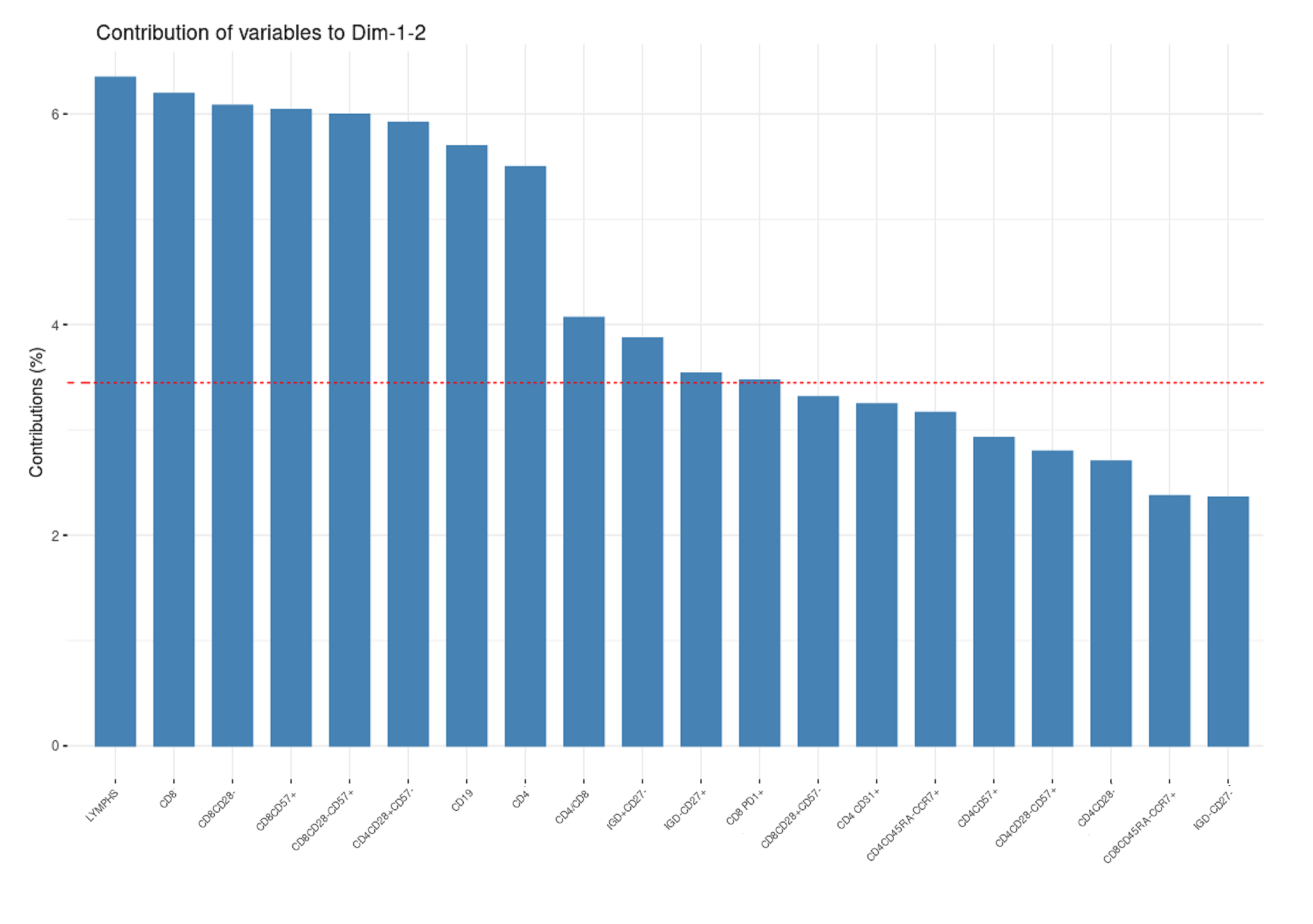

Supplement: Supplementary file 2 [file Image_2.png]

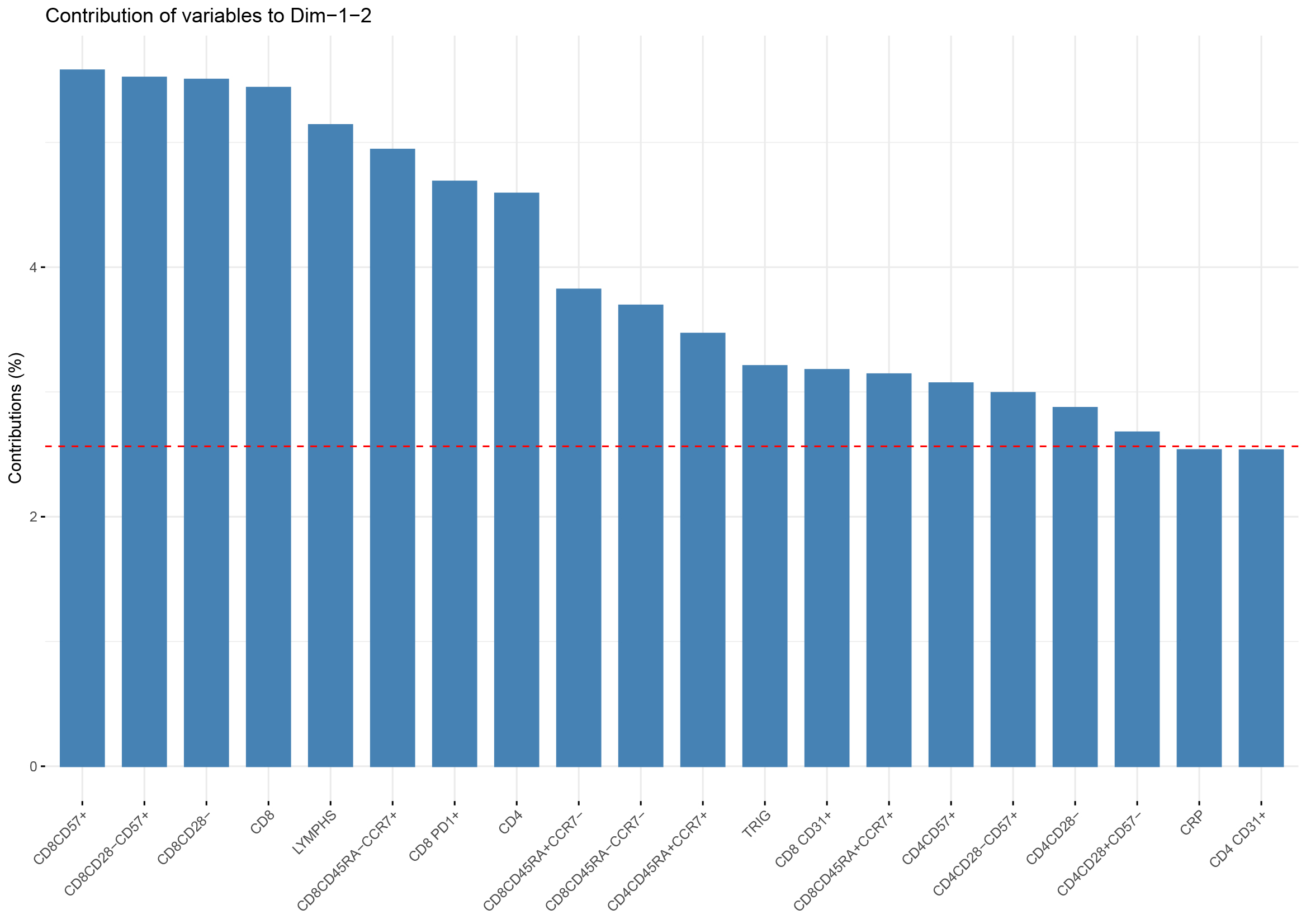

Supplement: Supplementary file 3 [file Image_3.jpeg]
