# Supplementary material for: Clustering of End Stage Renal Disease Patients by Dimensionality Reduction Algorithms According to Lymphocyte Senescence Markers
Source: Front Immunol. 2022 May 9;13:841031. doi: 10.3389/fimmu.2022.841031 (PMC9126282; doi:10.3389/fimmu.2022.841031)
Supplement: Supplementary file 4 [file Table_1.docx]

Supplementary Material

# Supplementary Table

**Table S1. Conjugated monoclonal antibodies and combinations applied**

| **Tube 1** | **Tube 2** | **Tube 3** | **Tube 4** |
| --- | --- | --- | --- |
| *T cell markers* | *T cell markers* | *T cell markers* | *B cell markers* |
| CD45 PC7 | CD45 PC7 | CD45 PC7 | CD45 PC7 |
| CD3 FITC | CD3 PE | CD3 FITC | CD19 PC5 |
| CD4 Pacific Blue | CD4 Pacific Blue | CD4 Pacific Blue | IgD FITC |
| CD8 PC5 | CD8 PC5 | CD8 PC5 | CD27 ECD |
| CD45RA APC | CD45RA APC | CD45RA APC |  |
| CCR7 PE | CD57 FITC | CD31 ECD |  |
| CD28 ECD | CD28 ECD | PD1 PE |  |

# Supplementary Figures

**Supplementary Figure 1.** **Gating strategy for CD8 T cells and B cells.** The same gating strategy as for CD8 T cells was also used for CD4 T cells.

**Supplementary Figure 2. Contribution of variables in dimensions 1 and 2 of PCA for patients and controls.**

**Supplementary Figure 3.** **Contribution of variables in dimensions 1 and 2 of PCA for patients.**
